# 6-Bromo-3-hydr­oxy-4-oxo-2-phenyl-4*H*-chromene-8-carboxylic acid dimethyl­formamide disolvate

**DOI:** 10.1107/S1600536808019454

**Published:** 2008-07-05

**Authors:** Hui-Liang Wen, Dan-Dan Chen, Chong-Bo Liu

**Affiliations:** a Department of Chemistry, Nanchang University, Nanchang 330031, People’s Republic of China; b State Key Laboratory of Food Science and Technology, Nanchang University, Nanchang 330047, People’s Republic of China; c College of Environmental and Chemical Engineering, Nanchang University of Aeronautics, Nanchang 330063, People’s Republic of China

## Abstract

In the title compound, C_16_H_9_BrO_5_·2C_3_H_7_NO, the chromene ring system is essentially planar. The two dimethyl­formamide solvent mol­ecules are linked by inter­molecular O—H⋯O hydrogen bonds to the 6-bromo-3-hydr­oxy-4-oxo-2-phenyl-4H-chromene-8-carboxylic acid molecules.

## Related literature

For related literature, see: Gills *et al.* (1980 ▶); Liu *et al.* (2007 ▶); Jin & Xiao (2005 ▶); Kagechika *et al.* (1989 ▶); Valenti *et al.* (1998 ▶); Walenta *et al.* (1991 ▶); Zwaagstra *et al.* (1996 ▶, 1998*a* ▶,*b* ▶).
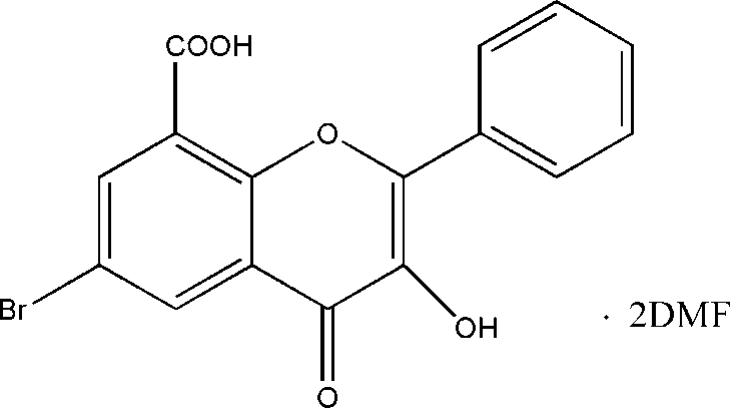


## Experimental

### 

#### Crystal data

C_16_H_9_BrO_5_·2C_3_H_7_NO
*M* *_r_* = 507.33
Monoclinic, 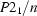

*a* = 10.489 (2) Å
*b* = 11.470 (2) Å
*c* = 18.803 (4) Å
β = 92.127 (3)°
*V* = 2260.6 (8) Å^3^
*Z* = 4
Mo *K*α radiation
μ = 1.86 mm^−1^
*T* = 294 (2) K
0.49 × 0.38 × 0.17 mm

#### Data collection

Bruker SMART CCD diffractometer
Absorption correction: multi-scan (*SADABS*; Sheldrick, 1996 ▶) *T*_min_ = 0.462, *T*_max_ = 0.742
14295 measured reflections
4203 independent reflections
2889 reflections with *I* > 2σ(*I*)
*R*_int_ = 0.028

#### Refinement

*R*[*F* ^2^ > 2σ(*F* ^2^)] = 0.036
*wR*(*F* ^2^) = 0.098
*S* = 0.99
4203 reflections
294 parameters
H-atom parameters constrained
Δρ_max_ = 0.32 e Å^−3^
Δρ_min_ = −0.34 e Å^−3^

### 

Data collection: *SMART* (Bruker, 1998 ▶); cell refinement: *SAINT* (Bruker, 1998 ▶); data reduction: *SAINT*; program(s) used to solve structure: *SHELXS97* (Sheldrick, 2008 ▶); program(s) used to refine structure: *SHELXL97* (Sheldrick, 2008 ▶); molecular graphics: *ORTEPIII* (Burnett & Johnson,1996 ▶); *ORTEP-3 for Windows* (Farrugia, 1997 ▶); software used to prepare material for publication: *SHELXL97*.

## Supplementary Material

Crystal structure: contains datablocks global, I. DOI: 10.1107/S1600536808019454/dn2358sup1.cif

Structure factors: contains datablocks I. DOI: 10.1107/S1600536808019454/dn2358Isup2.hkl

Additional supplementary materials:  crystallographic information; 3D view; checkCIF report

## Figures and Tables

**Table 1:** Hydrogen-bond geometry (Å, °)

*D*—H⋯*A*	*D*—H	H⋯*A*	*D*⋯*A*	*D*—H⋯*A*
O2—H2*A*⋯O6	0.82	1.78	2.598 (2)	173
O5—H5⋯O7	0.82	1.89	2.627 (2)	149
O5—H5⋯O4	0.82	2.32	2.741 (3)	113

**Fig. 1.**
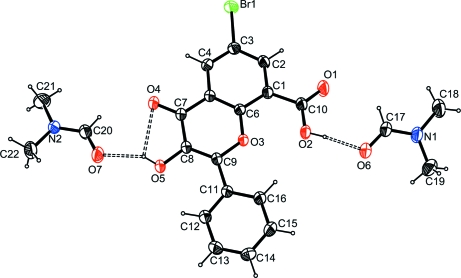
: The molecular structure of (I), with the atom-labelling scheme. Displacement ellipsoids are drawn at the 30% probability level and H atoms are represented as small spheres of arbitrary radii. The hydrogen bonds are shown as dashed lines.

## supplementary crystallographic information

### Comment

Flavonoids are widely present in nature, which have potential biological activity such as antiviral (Zwaagstra *et al.*, 1996; Zwaagstra *et al.*, 1998*a*), anticancer (Valenti *et al.*, 1998), treating leukemia (Kagechika *et al.*, 1989), antihypertensive, antimicrobial (Gills *et al.*, 1980; Walenta *et al.*, 1991) *et al.* Due to the varieties of its biological activity, the structure-activity relationships study of flavonoids carboxylic acids has been the hot spot all along. In a continuation of our recent studies of flavonoids carboxylic acids (Liu *et al.*, 2007), we report here the title compound, C_16_H_9_BrO_5_—C_6_H_14_N_2_O_2_, (I).

In compound (I), the chromene molecule is roughly planar, with a mean deviation of 0.0521 Å. The dihedral angle between the chromene ring and the phenyl ring is 7.5 (2)°. Two O—H···O hydrogen bonds (Table 1, Fig. 1) involving the H atoms of hydroxyl group and carboxylic acid group connect the dimethylformamide molecules and 6-bromo-3-hydroxy-4-oxo-2-phenyl-4*H*-chromene-8-carboxylic acid.

### Experimental

The title compound was synthesized by the ring closure of 5'-bromo-3'-carboxy-2'-hydroxychalcone under the existence of a certain oxidant, according to the route published by Zwaagstra *et al.* (Zwaagstra *et al.*, 1998*b*). Single crystals of (I) suitable for X-ray diffraction analysis were obtained from a solution in *N*,*N*-dimethylformamide.

### Refinement

All H atoms attached to C atoms and O atom were fixed geometrically and treated as riding with C—H = 0.96 Å (methyl) or 0.93 Å (aromatic) and O—H = 0.82 Å with U_iso_(H) = 1.2U_eq_(C_aromatic_) or U_iso_(H) = 1.5U_eq_(C_methyl_ and O).

### Crystal data

**Table:**

C_16_H_9_BrO_5_·2C_3_H_7_NO	*F*_000_ = 1040
*M**_r_* = 507.33	*D*_x_ = 1.491 Mg m^−^^3^
Monoclinic, *P*2(1)/*n*	Mo *K*α radiation λ = 0.71073 Å
Hall symbol: -P 2yn	Cell parameters from 3645 reflections
*a* = 10.489 (2) Å	θ = 2.6–23.5º
*b* = 11.470 (2) Å	µ = 1.86 mm^−^^1^
*c* = 18.803 (4) Å	*T* = 294 (2) K
β = 92.127 (3)º	Block, yellow
*V* = 2260.6 (8) Å^3^	0.49 × 0.38 × 0.17 mm
*Z* = 4	

### Data collection

**Table:**

Bruker SMART CCD diffractometer	4203 independent reflections
Radiation source: fine-focus sealed tube	2889 reflections with *I* > 2σ(*I*)
Monochromator: graphite	*R*_int_ = 0.028
*T* = 294(2) K	θ_max_ = 25.5º
φ and ω scans	θ_min_ = 2.6º
Absorption correction: multi-scan(SADABS; Sheldrick, 1996)	*h* = −12→11
*T*_min_ = 0.462, *T*_max_ = 0.742	*k* = −13→13
14295 measured reflections	*l* = −22→22

### Refinement

**Table:**

Refinement on *F*^2^	Secondary atom site location: difference Fourier map
Least-squares matrix: full	Hydrogen site location: inferred from neighbouring sites
*R*[*F*^2^ > 2σ(*F*^2^)] = 0.036	H-atom parameters constrained
*wR*(*F*^2^) = 0.098	*w* = 1/[σ^2^(*F*_o_^2^) + (0.0482*P*)^2^ + 0.6824*P*] where *P* = (*F*_o_^2^ + 2*F*_c_^2^)/3
*S* = 1.00	(Δ/σ)_max_ = 0.017
4203 reflections	Δρ_max_ = 0.32 e Å^−^^3^
294 parameters	Δρ_min_ = −0.34 e Å^−^^3^
Primary atom site location: structure-invariant direct methods	Extinction correction: none

### Special details

**Table:**

Geometry. All esds (except the esd in the dihedral angle between two l.s. planes) are estimated using the full covariance matrix. The cell esds are taken into account individually in the estimation of esds in distances, angles and torsion angles; correlations between esds in cell parameters are only used when they are defined by crystal symmetry. An approximate (isotropic) treatment of cell esds is used for estimating esds involving l.s. planes.
Refinement. Refinement of *F*^2^ against ALL reflections. The weighted *R*-factor *wR* and goodness of fit *S* are based on *F*^2^, conventional *R*-factors *R* are based on *F*, with *F* set to zero for negative *F*^2^. The threshold expression of *F*^2^ > σ(*F*^2^) is used only for calculating *R*-factors(gt) *etc*. and is not relevant to the choice of reflections for refinement. *R*-factors based on *F*^2^ are statistically about twice as large as those based on *F*, and *R*- factors based on ALL data will be even larger.

### Fractional atomic coordinates and isotropic or equivalent isotropic displacement parameters (Å^2^)

**Table:**

	*x*	*y*	*z*	*U*_iso_*/*U*_eq_	
Br1	0.06013 (3)	0.88028 (2)	0.582768 (19)	0.07113 (15)	
O1	−0.0368 (2)	0.44074 (18)	0.65009 (12)	0.0911 (8)	
O2	0.06856 (17)	0.31912 (16)	0.58290 (9)	0.0577 (5)	
H2A	0.0279	0.2704	0.6046	0.087*	
O3	0.24560 (16)	0.40314 (13)	0.50359 (9)	0.0460 (4)	
O4	0.41242 (18)	0.68075 (16)	0.41187 (10)	0.0652 (6)	
O5	0.49923 (18)	0.46087 (15)	0.38343 (11)	0.0610 (5)	
H5	0.5303	0.5237	0.3727	0.091*	
C1	0.1082 (2)	0.5216 (2)	0.57023 (13)	0.0439 (6)	
C2	0.0684 (2)	0.6327 (2)	0.58763 (14)	0.0517 (7)	
H2	0.0041	0.6416	0.6199	0.062*	
C3	0.1222 (2)	0.7312 (2)	0.55796 (14)	0.0495 (7)	
C4	0.2183 (2)	0.7207 (2)	0.51073 (14)	0.0485 (6)	
H4	0.2544	0.7869	0.4912	0.058*	
C5	0.2615 (2)	0.6101 (2)	0.49221 (13)	0.0422 (6)	
C6	0.2067 (2)	0.5112 (2)	0.52147 (12)	0.0411 (6)	
C7	0.3634 (2)	0.5972 (2)	0.44191 (14)	0.0456 (6)	
C8	0.4030 (2)	0.4787 (2)	0.42805 (13)	0.0443 (6)	
C9	0.3421 (2)	0.3864 (2)	0.45729 (13)	0.0429 (6)	
C10	0.0406 (3)	0.4222 (2)	0.60528 (14)	0.0518 (7)	
C11	0.3633 (2)	0.2608 (2)	0.44578 (13)	0.0442 (6)	
C12	0.4628 (3)	0.2186 (3)	0.40674 (19)	0.0821 (11)	
H12	0.5188	0.2708	0.3865	0.099*	
C13	0.4800 (4)	0.1012 (3)	0.3976 (2)	0.0944 (13)	
H13	0.5482	0.0752	0.3716	0.113*	
C14	0.3998 (3)	0.0217 (2)	0.42550 (17)	0.0686 (9)	
H14	0.4114	−0.0578	0.4184	0.082*	
C15	0.3021 (3)	0.0619 (2)	0.46419 (18)	0.0736 (9)	
H15	0.2464	0.0089	0.4839	0.088*	
C16	0.2840 (3)	0.1793 (2)	0.47476 (16)	0.0629 (8)	
H16	0.2169	0.2041	0.5020	0.075*	
N1	−0.2166 (3)	0.1136 (2)	0.71663 (13)	0.0631 (7)	
O6	−0.0563 (2)	0.15290 (18)	0.64329 (11)	0.0722 (6)	
C17	−0.1516 (3)	0.1828 (3)	0.67549 (16)	0.0626 (8)	
H17	−0.1792	0.2595	0.6703	0.075*	
C18	−0.3276 (4)	0.1567 (3)	0.7524 (2)	0.0983 (13)	
H18A	−0.3072	0.1649	0.8024	0.147*	
H18B	−0.3968	0.1026	0.7456	0.147*	
H18C	−0.3521	0.2311	0.7329	0.147*	
C19	−0.1781 (3)	−0.0070 (3)	0.72818 (18)	0.0840 (10)	
H19A	−0.2251	−0.0565	0.6955	0.126*	
H19B	−0.1953	−0.0294	0.7761	0.126*	
H19C	−0.0885	−0.0147	0.7206	0.126*	
N2	0.7254 (2)	0.78411 (18)	0.28823 (12)	0.0554 (6)	
O7	0.6435 (2)	0.60874 (17)	0.31631 (12)	0.0773 (7)	
C20	0.6539 (3)	0.7141 (3)	0.32545 (16)	0.0642 (8)	
H20	0.6076	0.7473	0.3615	0.077*	
C21	0.7365 (4)	0.9061 (3)	0.3036 (3)	0.1108 (15)	
H21A	0.6909	0.9239	0.3456	0.166*	
H21B	0.7012	0.9503	0.2642	0.166*	
H21C	0.8248	0.9260	0.3113	0.166*	
C22	0.8037 (3)	0.7385 (3)	0.23293 (16)	0.0746 (9)	
H22A	0.8853	0.7160	0.2532	0.112*	
H22B	0.8151	0.7974	0.1975	0.112*	
H22C	0.7626	0.6718	0.2114	0.112*	

### Atomic displacement parameters (Å^2^)

**Table:**

	*U*^11^	*U*^22^	*U*^33^	*U*^12^	*U*^13^	*U*^23^
Br1	0.0723 (2)	0.04462 (18)	0.0983 (3)	0.00670 (14)	0.02689 (18)	−0.00929 (15)
O1	0.1120 (19)	0.0564 (13)	0.1106 (18)	−0.0065 (12)	0.0794 (16)	−0.0021 (12)
O2	0.0641 (13)	0.0440 (11)	0.0672 (12)	−0.0095 (9)	0.0321 (10)	0.0013 (9)
O3	0.0491 (10)	0.0358 (9)	0.0546 (10)	−0.0028 (7)	0.0219 (8)	0.0004 (7)
O4	0.0696 (14)	0.0428 (11)	0.0860 (14)	−0.0029 (9)	0.0389 (11)	0.0106 (10)
O5	0.0621 (13)	0.0421 (10)	0.0814 (13)	−0.0017 (9)	0.0392 (10)	0.0058 (9)
C1	0.0440 (15)	0.0429 (14)	0.0454 (14)	−0.0045 (11)	0.0098 (12)	0.0000 (11)
C2	0.0483 (16)	0.0522 (16)	0.0557 (16)	−0.0010 (12)	0.0179 (13)	−0.0043 (12)
C3	0.0501 (16)	0.0402 (14)	0.0587 (17)	0.0007 (12)	0.0073 (13)	−0.0050 (12)
C4	0.0486 (16)	0.0375 (13)	0.0600 (17)	−0.0043 (11)	0.0085 (13)	0.0032 (12)
C5	0.0411 (14)	0.0389 (13)	0.0472 (14)	−0.0012 (11)	0.0078 (11)	0.0025 (11)
C6	0.0411 (14)	0.0372 (13)	0.0455 (14)	−0.0011 (11)	0.0080 (11)	−0.0015 (11)
C7	0.0425 (15)	0.0412 (14)	0.0540 (15)	−0.0033 (11)	0.0125 (12)	0.0060 (11)
C8	0.0413 (15)	0.0429 (14)	0.0494 (14)	−0.0025 (11)	0.0131 (12)	0.0040 (11)
C9	0.0401 (14)	0.0432 (13)	0.0460 (14)	−0.0022 (11)	0.0116 (11)	0.0022 (11)
C10	0.0528 (17)	0.0501 (15)	0.0537 (16)	−0.0041 (13)	0.0197 (14)	−0.0009 (12)
C11	0.0448 (15)	0.0383 (13)	0.0500 (15)	−0.0009 (11)	0.0102 (12)	−0.0001 (11)
C12	0.083 (2)	0.0446 (16)	0.123 (3)	−0.0012 (15)	0.061 (2)	0.0002 (17)
C13	0.099 (3)	0.0519 (19)	0.137 (3)	0.0085 (18)	0.070 (3)	−0.0067 (19)
C14	0.079 (2)	0.0396 (16)	0.089 (2)	0.0024 (15)	0.0210 (18)	−0.0071 (15)
C15	0.085 (2)	0.0397 (15)	0.099 (2)	−0.0087 (16)	0.0338 (19)	0.0043 (16)
C16	0.069 (2)	0.0440 (15)	0.078 (2)	−0.0023 (14)	0.0344 (16)	−0.0023 (14)
N1	0.0694 (17)	0.0582 (15)	0.0630 (15)	−0.0122 (12)	0.0213 (13)	0.0075 (11)
O6	0.0710 (15)	0.0588 (12)	0.0889 (15)	−0.0066 (10)	0.0320 (12)	0.0131 (11)
C17	0.064 (2)	0.0532 (17)	0.072 (2)	−0.0080 (15)	0.0209 (16)	0.0106 (15)
C18	0.096 (3)	0.088 (3)	0.114 (3)	−0.014 (2)	0.057 (2)	0.004 (2)
C19	0.104 (3)	0.064 (2)	0.084 (2)	−0.0043 (19)	0.016 (2)	0.0247 (18)
N2	0.0544 (14)	0.0404 (12)	0.0727 (15)	−0.0045 (10)	0.0200 (12)	0.0015 (11)
O7	0.0906 (17)	0.0485 (13)	0.0959 (16)	−0.0178 (10)	0.0453 (13)	−0.0037 (10)
C20	0.065 (2)	0.0550 (18)	0.075 (2)	−0.0054 (15)	0.0276 (16)	−0.0033 (15)
C21	0.114 (3)	0.0420 (18)	0.180 (4)	−0.0070 (19)	0.056 (3)	−0.007 (2)
C22	0.087 (2)	0.072 (2)	0.067 (2)	−0.0156 (17)	0.0320 (18)	−0.0041 (16)

### Geometric parameters (Å, °)

**Table:**

Br1—C3	1.894 (2)	C14—C15	1.359 (4)
O1—C10	1.210 (3)	C14—H14	0.9300
O2—C10	1.293 (3)	C15—C16	1.376 (4)
O2—H2A	0.8200	C15—H15	0.9300
O3—C6	1.351 (3)	C16—H16	0.9300
O3—C9	1.372 (3)	N1—C17	1.316 (3)
O4—C7	1.235 (3)	N1—C18	1.453 (4)
O5—C8	1.352 (3)	N1—C19	1.455 (4)
O5—H5	0.8200	O6—C17	1.236 (3)
C1—C2	1.384 (3)	C17—H17	0.9300
C1—C6	1.412 (3)	C18—H18A	0.9600
C1—C10	1.507 (3)	C18—H18B	0.9600
C2—C3	1.389 (4)	C18—H18C	0.9600
C2—H2	0.9300	C19—H19A	0.9600
C3—C4	1.373 (3)	C19—H19B	0.9600
C4—C5	1.396 (3)	C19—H19C	0.9600
C4—H4	0.9300	N2—C20	1.318 (3)
C5—C6	1.394 (3)	N2—C21	1.433 (4)
C5—C7	1.460 (3)	N2—C22	1.447 (3)
C7—C8	1.447 (3)	O7—C20	1.225 (3)
C8—C9	1.363 (3)	C20—H20	0.9300
C9—C11	1.475 (3)	C21—H21A	0.9600
C11—C16	1.377 (3)	C21—H21B	0.9600
C11—C12	1.386 (4)	C21—H21C	0.9600
C12—C13	1.371 (4)	C22—H22A	0.9600
C12—H12	0.9300	C22—H22B	0.9600
C13—C14	1.359 (4)	C22—H22C	0.9600
C13—H13	0.9300		
			
C10—O2—H2A	109.5	C13—C14—H14	121.0
C6—O3—C9	121.48 (18)	C14—C15—C16	121.4 (3)
C8—O5—H5	109.5	C14—C15—H15	119.3
C2—C1—C6	117.8 (2)	C16—C15—H15	119.3
C2—C1—C10	116.2 (2)	C15—C16—C11	121.2 (3)
C6—C1—C10	126.0 (2)	C15—C16—H16	119.4
C1—C2—C3	121.6 (2)	C11—C16—H16	119.4
C1—C2—H2	119.2	C17—N1—C18	120.6 (3)
C3—C2—H2	119.2	C17—N1—C19	120.9 (3)
C4—C3—C2	120.5 (2)	C18—N1—C19	118.5 (3)
C4—C3—Br1	120.38 (19)	O6—C17—N1	124.5 (3)
C2—C3—Br1	119.14 (19)	O6—C17—H17	117.7
C3—C4—C5	119.6 (2)	N1—C17—H17	117.7
C3—C4—H4	120.2	N1—C18—H18A	109.5
C5—C4—H4	120.2	N1—C18—H18B	109.5
C6—C5—C4	119.9 (2)	H18A—C18—H18B	109.5
C6—C5—C7	119.7 (2)	N1—C18—H18C	109.5
C4—C5—C7	120.4 (2)	H18A—C18—H18C	109.5
O3—C6—C5	121.0 (2)	H18B—C18—H18C	109.5
O3—C6—C1	118.3 (2)	N1—C19—H19A	109.5
C5—C6—C1	120.6 (2)	N1—C19—H19B	109.5
O4—C7—C8	121.3 (2)	H19A—C19—H19B	109.5
O4—C7—C5	123.0 (2)	N1—C19—H19C	109.5
C8—C7—C5	115.7 (2)	H19A—C19—H19C	109.5
O5—C8—C9	120.3 (2)	H19B—C19—H19C	109.5
O5—C8—C7	118.7 (2)	C20—N2—C21	122.1 (3)
C9—C8—C7	120.9 (2)	C20—N2—C22	120.7 (2)
C8—C9—O3	121.0 (2)	C21—N2—C22	117.0 (2)
C8—C9—C11	128.6 (2)	O7—C20—N2	125.2 (3)
O3—C9—C11	110.38 (19)	O7—C20—H20	117.4
O1—C10—O2	123.6 (2)	N2—C20—H20	117.4
O1—C10—C1	120.7 (2)	N2—C21—H21A	109.5
O2—C10—C1	115.7 (2)	N2—C21—H21B	109.5
C16—C11—C12	116.8 (2)	H21A—C21—H21B	109.5
C16—C11—C9	120.6 (2)	N2—C21—H21C	109.5
C12—C11—C9	122.7 (2)	H21A—C21—H21C	109.5
C13—C12—C11	121.0 (3)	H21B—C21—H21C	109.5
C13—C12—H12	119.5	N2—C22—H22A	109.5
C11—C12—H12	119.5	N2—C22—H22B	109.5
C14—C13—C12	121.6 (3)	H22A—C22—H22B	109.5
C14—C13—H13	119.2	N2—C22—H22C	109.5
C12—C13—H13	119.2	H22A—C22—H22C	109.5
C15—C14—C13	118.0 (3)	H22B—C22—H22C	109.5
C15—C14—H14	121.0		

### Hydrogen-bond geometry (Å, °)

**Table:**

*D*—H···*A*	*D*—H	H···*A*	*D*···*A*	*D*—H···*A*
O2—H2A···O6	0.82	1.78	2.598 (2)	173
O5—H5···O7	0.82	1.89	2.627 (2)	149
O5—H5···O4	0.82	2.32	2.741 (3)	113
